# Head and Neck Cancer Susceptibility and Metabolism in Fanconi Anemia

**DOI:** 10.3390/cancers14082040

**Published:** 2022-04-18

**Authors:** Tafadzwa Chihanga, Sara Vicente-Muñoz, Sonya Ruiz-Torres, Bidisha Pal, Mathieu Sertorio, Paul R. Andreassen, Ruby Khoury, Parinda Mehta, Stella M. Davies, Andrew N. Lane, Lindsey E. Romick-Rosendale, Susanne I. Wells

**Affiliations:** 1 Division of Oncology, Cincinnati Children’s Hospital Medical Center, Cincinnati, OH 45229, USA; tafadzwa.chihanga@cchmc.org (T.C.); sonya.ruiz.torres@gmail.com (S.R.-T.); bidisha.pal@cchmc.org (B.P.); 2 Department of Pathology and Laboratory Medicine, Cincinnati Children’s Hospital Medical Center, Cincinnati, OH 45229, USA; sara.vicentemunoz@cchmc.org (S.V.-M.); lindsey.romick-rosendale@cchmc.org (L.E.R.-R.); 3 Department of Radiation Oncology, College of Medicine, University of Cincinnati, Cincinnati, OH 45229, USA; sertormu@ucmail.uc.edu; 4 Division of Experimental Hematology and Cancer Biology, Cincinnati Children’s Hospital Medical Center, Cincinnati, OH 45229, USA; paul.andreassen@cchmc.org; 5 Division of Bone Marrow Transplantation and Immune Deficiency, Cincinnati Children’s Hospital Medical Center, Cincinnati, OH 45229, USA; ruby.khoury@cchmc.org (R.K.); parinda.mehta@cchmc.org (P.M.); stella.davies@cchmc.org (S.M.D.); 6 Department of Toxicology and Cancer Biology, University of Kentucky, Lexington, KY 40536, USA; andrew.lane@uky.edu

**Keywords:** Fanconi anemia, human papillomavirus, squamous cell carcinoma, metabolism, aldehydes, reactive oxygen species, mitochondria, lipids

## Abstract

**Simple Summary:**

Germline loss-of-function mutations in any of over 20 known Fanconi anemia (FA) genes cause the disorder FA. The FA pathway plays a key role in the repair of DNA interstrand crosslinks (ICLs) and stabilization of stalled replication fork. Epidemiological studies have shown that persons with FA have a uniquely increased susceptibility to squamous cell carcinomas (SCCs) and increased human papilloma virus (HPV) prevalence. Genomic and transcriptomic studies are informative, but there is a paucity of data regarding metabolic deregulation in FA deficient systems. Metabolism is an essential component of cancer development and has significant impact on therapeutic outcomes. Therefore, a deeper understanding of the metabolic architecture of FA may aid in our understanding of SCC development, and allow for the discovery of metabolism-based diagnostic biomarkers and new therapeutic targets. In this review, we discuss known metabolic consequences of FA pathway loss on cancer pathogenesis, and in particular in normal and transformed keratinocytes—the cells of origin for SCC.

**Abstract:**

Fanconi anemia (FA) is a rare inherited, generally autosomal recessive syndrome, but it displays X-linked or dominant negative inheritance for certain genes. FA is characterized by a deficiency in DNA damage repair that results in bone marrow failure, and in an increased risk for various epithelial tumors, most commonly squamous cell carcinomas of the head and neck (HNSCC) and of the esophagus, anogenital tract and skin. Individuals with FA exhibit increased human papilloma virus (HPV) prevalence. Furthermore, a subset of anogenital squamous cell carcinomas (SCCs) in FA harbor HPV sequences and FA-deficient laboratory models reveal molecular crosstalk between HPV and FA proteins. However, a definitive role for HPV in HNSCC development in the FA patient population is unproven. Cellular metabolism plays an integral role in tissue homeostasis, and metabolic deregulation is a known hallmark of cancer progression that supports uncontrolled proliferation, tumor development and metastatic dissemination. The metabolic consequences of FA deficiency in keratinocytes and associated impact on the development of SCC in the FA population is poorly understood. Herein, we review the current literature on the metabolic consequences of FA deficiency and potential effects of resulting metabolic reprogramming on FA cancer phenotypes.

## 1. Introduction

Fanconi anemia (FA) is a generally autosomal recessive orphan disease caused by germline loss of function mutations in any one of more than 20 genes (Table 1) associated with the FA DNA repair pathway, although rare cases display X-linked or dominant negative inheritance [1]. FA affects 1 in 160,000 individuals, with a variable life expectancy of 20 years [2,3]. The FA pathway functions classically in the repair of DNA (interstrand crosslinks) ICLs [1]. ICLs are covalent adducts between DNA strands which block transcription and replication and can lead to double strand DNA breaks [4]. Continuous removal of ICLs is required for sustained cell survival. ICLs form as a response to either exogenous or endogenous crosslinkers. Exogenous crosslinkers include chemotherapeutics such as mitomycin C, cisplatin, melphalan, psoralens, tobacco use, broiled meat and alcohol consumption [5,6,7]. Endogenous crosslinkers include natural byproducts of mitochondrial and fat metabolism, metabolism of tobacco smoke or ingested alcohol, all of which can produce reactive oxygen species (ROS) [8] and aldehydes [9,10]. Cells from individuals with FA are highly sensitive to DNA crosslinkers, resulting in increased chromosomal abnormalities and accumulation of cells arrested in the G2/M phase of the cell cycle [5,11]. Individuals with FA are unable to properly repair ICLs, and exhibit congenital defects and progressive bone marrow failure, often early in life. Moreover, patients with FA demonstrate increased susceptibility to human papillomavirus (HPV) and SV40 infection [12,13]. Bone marrow failure in FA can evolve into leukemia [9,14,15,16,17,18] and persons with FA have a markedly elevated frequency of early aggressive keratinocyte-based squamous cell carcinomas (SCCs) of the head and neck, esophagus, anogenital tract, and skin with advancing age compared to the general population [1,19,20,21,22,23,24,25]. Other non-canonical activities reported for the FA pathway include the stabilization of stalled replication forks [26], control of mitosis and cytokinesis [27,28], suppression of non-homologous end-joining (NHEJ) [29], clearance of damaged mitochondria by mitophagy [30], clearance of viruses by autophagy [30], regulation of the HPV life cycle [13,21,24,31,32,33,34,35], maintenance of cellular redox reactions [36] and metabolic regulation [37,38,39]. In this review we address canonical and non-canonical activities of the FA machinery and pathological phenotypes that result from the loss of this pathway and describe metabolic consequences of aberrant FA pathway activity, and mechanisms whereby these can establish a pro-inflammatory and pro-oncogenic environment.

## 2. Molecular Components of the FA Machinery

**Figure 1:**
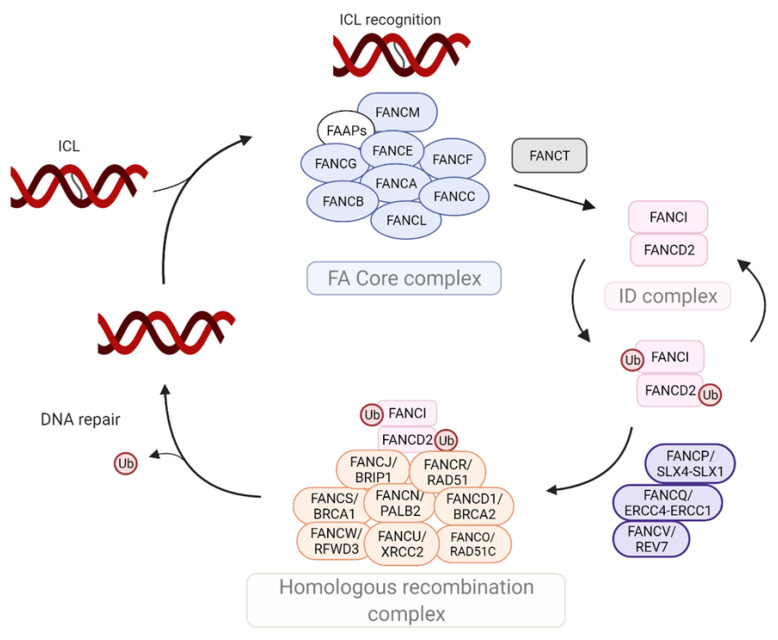
The Fanconi anemia (FA) DNA repair pathway specializes in the repair of DNA interstrand cross-links (ICLs). The FA pathway is activated in response to ICL formation. ICLs are recognized by the FANCM protein, which subsequently recruits the FA core complex to chromatin. The FA core complex is a ubiquitin E3 ligase composed of eight FA proteins (FANCA, FANCB, FANCC, FANCE, FANCF, FANCG, FANCL, FANCM; blue circles) as well as FA associated proteins (FAAPs) (white circle). The FA core complex monoubiquitinates the FANCD2:FANCI complex (pink rectangles) in conjunction with the E2 conjugating enzyme FANCT (green rectangle). The activated FANCI:FANCD2 (ID) complex then allows the recruitment of translesion synthesis and homologous recombination complex proteins (orange and purple ovals) to complete DNA repair. Silver spiral strand: interstrand cross link in the DNA double helix. Arrow: activation. FANCS/BRCA1: Breast cancer susceptibility protein 1; FANCN/PALB2: Partner and localizer of BRCA2; FANCJ/BRIP: BRCA interacting protein; FANCW/RFWD3: ring finger and WD repeat domain protein; FANCU/XRCC2: X-ray repair cross complementing protein; FANCP/SLX4: structure-specific endonuclease subunit; FANCD1/BRCA2: Breast cancer susceptibility protein 2; FANCO/RAD51C: Fanconi anemia, complementation group O; FANCR/RAD51: Fanconi anemia, complementation group R; FANCV/REV7: Fanconi anemia, complementation group V; FANCQ/ERCC4: Excision repair, complementing defective, in Chinese hamster, 4. The Fanconi anemia (FA) DNA repair pathway specializes in the repair of DNA interstrand cross-links (ICLs). Available online: https://biorender.com/illustrations (accessed on 11 March 2022).

FA pathway canonical activity relies on the function of more than 20 currently known FA proteins that repair ICLs in a highly coordinated fashion (Figure 1). FANCA (60%), FANCC (12%) and FANCG (XRCC9) (10%) are the most frequently mutated genes [1]. The FA pathway is subdivided into three main complexes (Table 1). FANCM, together with other FA-associated (FAA) proteins [40,41,42,43,44], recognizes an ICL and activates the ataxia telangiectasia and RAD3-related (ATR) and checkpoint kinase 1 (Chk1)-dependent checkpoint response [45,46], followed by the recruitment of the FA core complex to sites of DNA damage [41,47,48]. The FANCI/FANCD2 (ID) complex is then activated upon ATR phosphorylation of FANCI, via monoubiquitination by the FA core complex mediated through the E3 ligase activity of FANCL, thereby allowing FANCI heterodimerization with FANCD2 and recognition of ICLs [49,50,51]. The E3 ligase activity of FANCL requires FANCT (UBE2T) [52]. Importantly, inactivation of any of the FA core complex components inhibits ID complex activation, leaving the pathway non-functional [52,53,54]. Monoubiquitinated FANCD2 recruits endonucleases including FANCP (SLX4), SLX1, FANCQ (also known as ERCC4), ERCC1, MUS81-EME1 and FAN1 [55,56,57,58] as well as the exonuclease SNM1B [59]. These nucleases create incisions on either side of the ICL, allowing the unhooking of the monoadduct from one of the DNA strands. FANCD2 then recruits the translesion synthesis (TLS) polymerases, Pol η, Pol ν and the Pol ζ subunits Rev3, FANCV (Rev7) and Rev1, to fill the gap in the DNA strand, thus creating a template strand for homologous recombination to complete the repair [60,61,62].

## 3. Clinical Phenotypes in FA

FA proteins are expressed in every tissue [63] but the consequences of FA pathway loss are organ-specific. Relevant mechanisms are poorly understood, in part because most mouse models do not fully recapitulate the range of FA phenotypes [64]. A clinical hallmark of FA is progressive bone marrow failure, which is the result of hematopoietic stem cell exhaustion caused by stressors such as excessive TNFα levels [16], aldehyde toxicity [10] and/or overactive p53 signaling [65]. Bone marrow failure in FA can lead to the development of myelodysplastic syndrome (MDS) and acute myeloid leukemia (AML) [16,17,48]. The overall risk of AML and MDS is increased by 700- and 6000-fold, respectively, in individuals with FA mutations, compared to the general population [66]. Interestingly, acquired mutations and epigenetic silencing of FA genes have also been identified in sporadic AML, albeit at low frequencies [15,18,67,68]. Currently, the only curative therapeutic option for FA-related hematologic malignancies is hematopoietic stem cell transplantation (HSCT). Modified HSCT conditioning regimens using reduced doses of alkylating agents have improved survival for FA patients with hematological malignancies, although outcomes are still sub-optimal compared with the excellent results seen in non-FA individuals transplanted for marrow failure. Patients surviving after successful HSCT remain at a high risk for developing non-hematological malignancies [4,19,69]. In contrast to the bone marrow failure seen in the hematopoietic system, a similar decline in stem cell fitness in other hyper-proliferative organs such as the skin or mucosa has not been reported in FA. Instead, affected individuals are predisposed to early onset and highly aggressive keratinocyte based squamous cell carcinomas (SCCs) of the head and neck, esophagus, anogenital tract [19,20,21,70] and skin [71]. Indeed, the estimated risk of developing head and neck SCC, esophageal SCC and vulvar SCC in FA patients are increased 700-fold, 2000-fold and 4000-fold, respectively, relative to the general population [19,24,48,72]. Given the extreme sensitivity of FA-deficient cells to DNA damage and particularly crosslinks, conventional chemotherapy and radiation treatments in these patients are challenging, making surgical resection the preferred treatment option, and contributing to high tumor recurrence and dismal long-term survival [73].

There are few studies of epidermal carcinogenesis in individuals with FA. However, analysis of data from The Cancer Genome Atlas (TCGA) reveals that 18% of sporadic head and neck SCCs harbor point mutations and other variations in FA genes such as deletions [74], suggesting selective pressure for FA pathway loss during carcinogenesis. These results were further supported by recent transcriptome-wide association studies where reduced FANCA expression in sporadic skin SCC was identified [75]. FA-proficient, HNSCC-derived cell lines with knockdown of either FANCA, FANCD2 or FANCJ display FA-specific defects, a shift to an EMT-like state and a more invasive phenotype [76]. Together, these data suggest that constitutional FA pathway loss in the FA patient and acquired somatically in the general population supports early and aggressive SCC development in the skin and mucosa by currently poorly understood mechanisms. Furthermore, the skin of persons with FA, and the skin and mucosa of FA laboratory models, harbor defects in keratinocyte cell–cell and cell–substrate adhesion complexes, as well as EMT-like phenotypes [77]. Taken together with the finding that FA patients are susceptible to skin blistering, these results suggest that the FA pathway stabilizes epidermal integrity [77].

## 4. Dysregulation of Mitochondrial Metabolism in FA-Deficient Cells

FA pathway deficiency is associated with dysregulated cellular metabolism in addition to defects in ICL repair, and metabolic abnormalities are an important potential contributor to the observed clinical phenotypes. Metabolic dysregulation is reflected by changes in mitochondrial structure and function with reduced energy production and an increase in oxidative stress and defective mitophagy. Furthermore, mutations in FA proteins cause increased aldehyde load and subsequent aldehyde induced damage, resulting in reduced cellular capacity for aldehyde detoxification, and hyperproduction of, and sensitivity to, inflammatory cytokines [78,79]. Metabolic reprogramming in individuals with FA may be linked to an array of phenotypes poorly explained by deficient DNA repair, including short stature, insulin resistance, thyroid dysfunction, abnormal body mass index (BMI) and dyslipidemia [80].

Metabolism is a dynamic process which is essential for cell viability, from maintaining membrane potentials, provision of metabolic energy in the form of ATP via oxidation of nutrients (catabolism) for cell maintenance and repair, to cell proliferation that requires ATP to drive the formation of complex macromolecules (anabolism), and tissue specific activities such as contraction of muscle and generation of action potentials in the brain. Nutrient uptake and utilization are commonly altered in cancers [75] and many show a strong dependence on glutamine [76,77]. In FA cancers, mitochondrial defects lead to reduced oxygen consumption and TCA cycle activity, and a reduced dependence on glutaminolysis and Gln oxidation [36]. However, the amido N of Gln is required for formation of nucleobases and thus to support proliferation [78]. FA cell deficient in FANCC showed several amino acids including Gln were associated with ageing, whereas Asp and Glu were associated with cancer [81]. Thus, unless FA cells can upregulate glutamine synthetase, they will have an absolute dependence on exogenous Gln for proliferation. A known hallmark of cancer is uncontrolled proliferation, which is supported by the activation of pathways related to coupling energy production to biosynthesis. In this section we discuss a subset of relevant metabolic reprogramming mechanisms that frequently occur in cancer, namely ROS generation and mutations in mitochondrial DNA, aldehyde clearance and lipid metabolism. We compare and contrast the status of these processes in FA. A classic example of metabolic reprogramming that occurs in malignant cells but is largely absent in untransformed cells, is the “Warburg effect”, wherein lactic fermentation is the preferred metabolic process for glucose oxidation even under normoxic conditions, resulting in the accumulation and excretion of lactate [82,83,84]. The underlying molecular mechanisms leading to the Warburg effect continue to be refined and updated. Mitochondrial dysfunction due to mitochondrial DNA (mtDNA) mutations, accumulation of ROS and release of oncometabolites into the cytosol (a process known as retrograde signaling), has been shown to trigger cytosolic signaling pathways which promote neoplastic transformation [85].

### 4.1. Mitochondrial Activities Play a Role in Oncogenesis

Mitochondria are cellular organelles responsible for oxygen-dependent energy metabolism and are a main source and target of ROS formation. The transport of ADP, phosphate and protons across the inner membrane normally accelerates the rate of electron transport and most of the oxygen consumed by the respiratory electron chain is reduced to water. mtDNA encodes some but not all of the respiratory enzyme subunits that are essential for oxidative phosphorylation and rRNA and tRNA needed for mitochondrial protein synthesis. Nuclear DNA encodes the remaining mitochondrial proteins [86]. Mutations in mtDNA are thought to significantly stimulate oxidative phosphorylation, support neoplastic transformation, and fulfill the sustained bioenergetic demands of cancer cells [87]. Meta-analysis of 20 different cancer types from 859 non-FA patients showed that 66% harbored mutations in mtDNA [88]. Included in this analysis were adult leukemia (9/24) 38% and head and neck cancers (337/467) 72%, which harbored mutations in mtDNA [88]. Although the role of these mutations in cancer and metastasis remains unknown, they may increase energy metabolism and ROS generation and support cell survival. Several studies have uncovered links between FA gene products and mitochondrial dysfunction [38,89,90,91,92,93,94], and oxidative stress-derived mitochondrial dysfunction in combination with decreased scavenging of endogenous aldehydes [10], increased lipid peroxidation [95] and impaired ATP production [38,90,96], have emerged as metabolic phenotypic hallmarks of FA [90]. More detailed FA metabolic studies are needed to determine required compensatory reprogramming such as increased lactic fermentation which provides metabolic energy, intermediates for proliferative anabolism, and more potent tumor-supporting microenvironments by acidification and negative regulation of immune cell function [97].

FA has recently been identified as a mitochondrial disease (MD) given these and other connections between FA proteins and impaired mitochondrial activities [98]. MDs are a group of disorders associated with mutations in nuclear and mitochondrial DNA and consequent impaired oxidative phosphorylation. MDs are characterized by a number of clinical pathologies including short stature, exercise intolerance and hypertrophic or dilated cardiomyopathy [99]. Although FA shares limited clinical similarities with MDs aside from short stature, mitochondrial dysfunction is a hallmark in both instances [98].

### 4.2. FA Proteins Localize to Mitochondria

**Figure 2:**
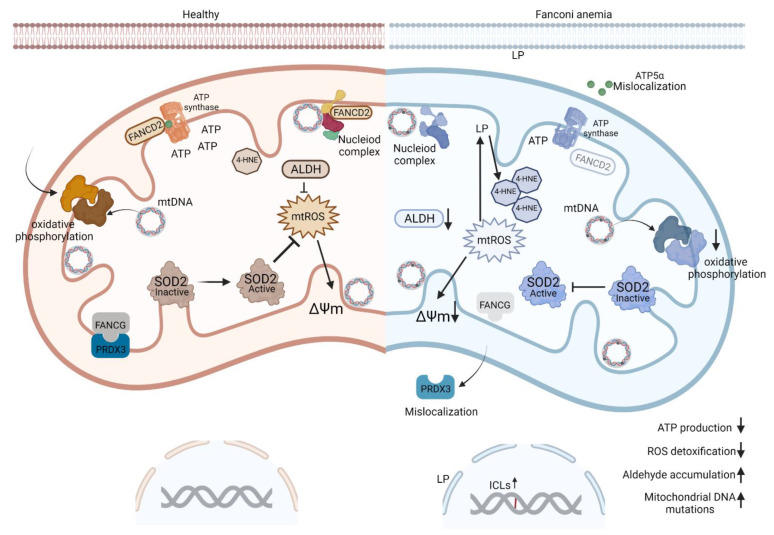
Schematic of Fanconi Anemia (FA) protein function in healthy (brown, left) versus FA-deficient (blue, right) mitochondria. Mitochondrial ROS (mtROS) are continuously generated by metabolic and inflammatory reactions. Mitochondrial DNA (mtDNA) primarily encodes mitochondrial proteins, including those involved in respiration and oxidative phosphorylation. Increased mtROS production leads to increased mtDNA mutations and lipid peroxidation (LP). FANCD2 localizes, in part, to mitochondria in a process facilitated by the ATPase Family AAA Domain-Containing Protein 3A (ATAD3) member (yellow barbell) of the mitochondrial nucleoid complex, which is essential for transcription and translation of mitochondrial proteins; FANCD2 deficiency destabilizes the nucleoid complex. FANCD2 also interacts with the ATP5α subunit (green dots) of ATP synthase. FAND2 knockdown results in mislocalization of ATP5α, and diminishes ATP production. In contrast, FANCG binds to PRDX3 on the inner mitochondrial membrane, and FANCG-mutant fibroblasts harbor mislocalized PRDX3, which results in reactive oxygen species (ROS) accumulation. Similar to peroxidase peroxiredoxin-3 (PRDX3), superoxide dismutase 2 (SOD2) has antioxidant activities in the mitochondria and is activated in the presence of ROS. FANCA-deficient cells have shown a decrease in SOD2 activity [104]. Uncontrolled accumulation of ROS results in decreased mitochondrial transmembrane potential (ΔΨm) and thus ATP synthesis. Schematic of FA protein function in healthy (brown, left) versus FA-deficient (blue, right) mitochondria. Available online: https://biorender.com/illustrations (accessed on 11 March 2022).

Multiple FA proteins are detectable in mitochondria and regulate mitochondrial metabolic function through physical interactions with other mitochondrial proteins, such as the peroxidase peroxiredoxin-3 (PRDX3) [91], and ATP synthase (ATP5α), a subunit of the mitochondrial ATPase (Figure 2). In FANCA, C and G deficient cells, constitutive oxidative stress suppressed mitochondrial activities by reducing the transmembrane potential, oxygen consumption rate, ATP production, and ROS detoxification [91]. FANCA and FANCC deficient subtypes also suppressed PRDX3, a member of a family of antioxidant enzymes which regulate physiological levels of hydrogen peroxide (H_2_O_2_) [91]. FANCG physically interacts with PRDX3, and FANCG mutated cells harbored a distorted mitochondrial structure and reduced thioredoxin-dependent peroxidase activity [91]. Overexpression of PRDX3 restored the resistance of FANCG-deficient cells to oxidative stress, while PRDX3 downregulation increased sensitivity to mitomycin C. FANCA and C deficient subtypes also harbored decreased PRDX3 expression, indicating an as of yet unknown functional interaction between other FA proteins and PRDX3 [91] (Figure 2). Furthermore, a physical interaction between FANCD2 and ATP5α was reported to be essential for optimal ATP synthesis [39]. FANCD2 deficient cells harbored reduced mitochondrial ATP production due to inappropriate ATP5α localization [39] (Figure 2). In addition, FANCD2 localizes to mitochondria in a process mediated by the ATPase Family AAA Domain-Containing Protein 3A (ATAD3) [100], a member of the mitochondrial nucleoid complex that also includes Mitochondrial Transcription Factor A (Tfam) and Mitochondrial Tu Translation Elongation Factor (Tufm). This complex is essential for mtDNA-encoded gene transcription, translation and mitochondrial biosynthesis [100,101,102] (Figure 2), and might therefore play a key role in the maintenance of mitochondrial activities. Indeed, genetic deletion of Fancd2 in murine hematopoietic stem and progenitor cells (HSPCs) led to a significant increase in mitochondrial number, mitochondrial protein synthesis, enzyme activity of mitochondrially encoded respiratory complexes and, consequently, OXPHOS and mtROS levels in HSPCs [100]. Increased mitochondrial number and proteins may cause an imbalance in nuclear and mitochondrially encoded factors that is further exacerbated by a significant increase in mitochondrial stress related proteins and a deregulated mitochondrial stress response in FANCD2 -deficient cells [100,103].

### 4.3. ROS May Be a Cause or Consequence of Mitochondrial Abnormalities in FA

ROS are produced by several endogenous sources that require oxygen [105]. H_2_O_2_ is generated by a wide variety of oxygen-dependent oxidation reactions, as well as by dismutation of superoxide [106]. Oxygen radicals, which include superoxide, oxidize macromolecules such as lipids and proteins, and can generate adducts between DNA strands [107]. Some of these negative effects can be countered by antioxidant defenses such as upregulation of the antioxidant detoxification components NADPH quinone oxidoreductase-1 and Redox factor-1 (Ref-1) [105,108]. Thus, oxidative stress is defined by the imbalance between oxidants and antioxidants [95,109]. The mitochondrial respiratory chain is the major producer of ROS, as a byproduct of ATP production facilitated by NADH oxidases [110]. Other endogenous enzymatic reactions not limited to the mitochondria which produce ROS are prostaglandin synthesis, phagocytosis, and the cytochrome P450 system [111]. In addition, exogenous agents such as metals, therapeutic agents, radiation and environmental toxins such as benzo(a)pyrene also produce ROS [111,112].

It has been suggested that mitochondria in FA deficient cells are shifted to a semi-resting state, where ATP production is defective and the rate of oxygen consumption is decreased due to compromised Complex I activity [90,113]. FANCA-deficient cells displayed defects in mitochondrial respiratory chain Complex I activity, resulting in diminished ATP production [113]. Impaired oxygen consumption and reduced mitochondrial membrane potential as a consequence of low ATP production have been identified as phenotypes of FANCA, FANCC and FANCD2 deficient cells [90]. Overexpression of superoxide dismutase 1 (SOD1) rescued oxygen uptake and respiration capacity in FA cells. Some mitochondrial enzymes responsible for ROS clearance were unable to respond to H_2_O_2_ in FA cells, suggesting impairment of the mitochondrial detoxifying machinery [90]. Indeed, in pathological conditions such as FA, buildup and/or failure to detoxify ROS further diminish mitochondrial activities [109,114,115]. Accumulation of ROS dissipates the transmembrane potential, which leads to lower ATP levels in FA deficient cells as compared to their genetically corrected counterparts. The low transmembrane potential and overproduction of ROS could be a result of alterations in mitochondrial morphology, including membrane thinning or rupture, abnormal shapes and mitophagy in FA cells. Treatment of the same cells with H_2_O_2_ further increased mitochondrial fragmentation. In turn, addition of the ROS scavenger N-acetyl-cysteine (NAC) lowered the production of ROS in FA-depleted cells [90]. Importantly, the sensitivity of FA deficient cells to crosslinking agents such as mitomycin C was reduced in the presence of ROS scavengers. Pretreatment of FA deficient cells with NAC significantly improved ATP production, restored oxygen consumption and stimulated increased resistance to MMC, albeit in the absence of rescue of abnormal mitochondrial morphologies [90]. These data suggest that some of the mitochondrially induced ROS-related phenotypes of FA deficient cells are reversible [90,113].

### 4.4. Increased ROS Exacerbate DNA Damage in FA

ROS at low concentrations can have beneficial roles in signal transduction [116,117] and organismal defense against pathogens [118,119]. However, ROS at high concentrations can induce oxidation and damage of DNA, protein and lipid, with subsequent pro-inflammatory cytokine production, apoptosis, autophagy and/or necrosis. Elevated ROS also induces chronic inflammation which promotes cancer initiation and may further increase cancer risk [120,121]. Chronic inflammation and infection (e.g., persistent HPV infection) can cause the production of chemokines and cytokines, which in turn promote cellular transformation [122], by-pass of tumor suppressor activity [123] and proliferation and angiogenesis [124,125]. ROS stimulates pro-inflammatory chemokine and cytokine production, which initiates a positive feedback loop resulting in further ROS accumulation and an inflammatory oncogenic environment [126]. Interestingly, ROS-dependent lipid peroxidation results in aldehyde production, especially malondialdehyde (MDA) and 4-hydroxynonenal (4HNE) (Figure 2), both of which stimulate ICLs and hyper-mutagenicity [117,127]. Heightened endogenous aldehyde production via ROS and lipid peroxidation may therefore co-operate with the classical ICL repair defects to produce structural variants and chromosomal abnormalities that are hallmarks of FA [128,129].

Damaged mitochondria in FA cells are also more likely to rupture, inducing apoptosis at lower levels of stress than in normal cells. The FANCA, FANCC, FANCD2, FANCF, FANCL, BRCA1/FANCS and BRCA2/FANCD1 proteins aid in Parkin-mediated mitophagy, implicating this non-canonical role of FA proteins in disease pathologies [30,93]. Defective mitophagy, in turn, leads to the accumulation of damaged mitochondria and concomitantly increased intracellular oxidative stress [130]. Interestingly, in murine embryonic fibroblasts (MEFs), FANCC is essential for host immunity against herpes simplex virus type 1 mutant strain and Sindbis virus and plays a role in virophagy as well as autophagy [30]. Overall, these data suggest that genetic FA defects lead to the accumulation of ROS, as well as mitochondrial abnormalities with impaired antioxidant defenses [131], thus further damaging mitochondria to diminish cellular respiration and ATP synthesis.

## 5. Metabolic Dysregulation in FA

### 5.1. Aldehydes Are Relevant DNA Crosslinkers in Fanconi Anemia

The FA DNA repair pathway plays a fundamental role in protecting cells against aldehyde-mediated ICLs [132,133]. Aldehydes are present in the environment, e.g., ingested in alcoholic beverages or fried food, and are generated from multiple endogenous processes such as demethylation reactions, ethanol, methanol, methylamine and adrenaline metabolism, lipid peroxidation and the oxidative breakdown of folates [134]. Aldehydes can be carcinogenic, and the accumulation of aldehyde species is controlled at multiple levels. The first tier of protection is clearance by aldehyde dehydrogenases (ALDHs) and the second tier is repair of aldehyde-induced DNA crosslinks by the DNA repair pathway typically deficient in patients with FA. The aldehyde dehydrogenase (ALDH) superfamily comprises 19 nicotinamide adenine dinucleotide (NAD^+^) or (NADP^+^)-dependent enzymes that regulate embryonic development, cell proliferation, differentiation and other biological processes.

The main function of ALDHs is to detoxify endogenous and exogenous aldehydes to non-toxic carboxylic acids. For example, acetaldehyde, which is generated by the oxidation of ethanol, is converted to acetic acid by ALDH2 and ALDH1A3 [135,136]. Acetic acid can support the tricarboxylic acid cycle and acetylation reactions as it is incorporated into acetyl coenzyme A by acetylCoA synthetases [137,138,139]. ALDH1A3, along with ALDH3A1, ALDH3B1 and ALDH3B3, participates in amino acid metabolism by converting the intermediate aldehyde forms of histidine (methylimidazole acetaldehyde), phenylalanine (phenyl-acetaldehyde), alanine (β-aminopropion-aldehyde) and tyrosine (3-methoxy-4-hydroxyphenyl acetaldehyde, 3,4-hydroxy-phenyl acetaldehyde, 3,4-hydroxy-mandelaldehyde and 3-methoxy-4-hydroxy-phenyl-glycoaldehyde) to their corresponding acids [138]. Other metabolic functions of these enzymes include the metabolism of lipid peroxidation byproducts [140], retinoic acid [141] and alcohol via cytochrome P450 enzymes [137,142]. ALDH1A3 specifically is dysregulated in multiple cancers, with high expression in pancreatic [143], gynecologic (ovarian [144], endometrial [145] and cervical [146]) cancers, and high-grade gliomas (HGGs) [147] via increased gene promoter methylation. However, low expression of ALDH1A3 is associated with non-small cell lung cancer (NSCLC), non-muscle invasive bladder cancer (NMIBC), and prostate cancer [146,148]. In head and neck squamous cell carcinoma [149], breast, and prostate cancer stem cells, increased ALDH levels act as ROS scavengers and confer chemotherapy and radiation resistance [150], supporting recurrence and/or secondary tumor formation.

Accumulation of aldehydes such acetaldehyde and formaldehyde exacerbate both ICL formation and protein modification and detoxification by ALDH2 and ADH5 [136], respectively, are primary clearance mechanism of these aldehydes [151]. Exposure of cells to formaldehyde destabilizes the FA protein, BRCA2 and formaldehyde may therefore increase ICL accumulation concomitant with suppressing DNA repair [152]. ALDHs therefore act as modifiers of FA phenotypes. The role of ALDH genotype as a modifier of the FA phenotype is illustrated by a study of Japanese patients with FA where the dominant negative ALDH2_rs671 allele genotype was linked to accelerated and early bone marrow failure [153,154]. Hematopoietic stem cells (HSCs) in FA patients are not only reduced in number and function, but are also progressively eliminated due to the accumulation of unrepaired DNA damage. These data suggest that increased levels of aldehyde, in the absence of scavengers such as ALDH, further damage already impaired hematopoietic stem cell function. Aldehydes may establish a positive feedback cycle, further stimulating genome instability in already FA-deficient cells and promoting stem cell loss and progression of marrow failure. It should be noted that the identity of specific aldehydes which might be responsible for the phenotypes observed in these studies is unknown due to the relative lack of reliable methods to identify and quantify aldehydes in organisms.

Joint knockout of the Aldh2 and Fancd2 genes in mice was shown to result in bone marrow failure and elevated cancer development [9,10]. Furthermore, transfer of embryos into wild type as opposed to knockout mothers was sufficient to reverse embryonic lethality, but pups were still born with a severely depleted hematopoietic stem and progenitor cell compartment [9,10]. In mice lacking both formaldehyde clearance enzymes Aldh2 and Aldh5, perinatal lethality, growth failure, lymphopenia and lymphoid malignancies were observed. These studies also discovered seven families with defects in ALDH2 and ALDH5, and a new bone marrow failure syndrome that is solely driven by formaldehyde accumulation. The authors hypothesized that suppressing endogenous formaldehyde accumulation could become a primary treatment strategy for both ALDH-related bone marrow syndromes as well as FA. These data support key contributions of both maternal and fetal Aldh2 to the preservation of genome integrity and subsequent FA-related pathologies such as cancer development and bone marrow failure.

### 5.2. Dysregulated Lipid Metabolism in FA Promotes Hyperproliferation and SCC Phenotypes

Susceptibility of FA patients to SCC of the head and neck, esophagus, anogenital tract and skin is poorly understood. There is an urgent need to identify FA-associated SCC biomarkers and to gain a better understanding of relevant underlying metabolic mechanisms that drive or sustain SCCs in individuals with FA. De-regulated lipid metabolism remains a prominent hallmark of cancer with significant implications for cancer prevention and treatment. Lipid metabolism involves the synthesis, storage and degradation of lipids, which comprise a diverse range of compounds [155,156]. Lipids carry out numerous functions within the cell, including cell membrane formation, energy storage, and epigenetic regulation [157]. Membrane formation requires a diverse group of lipids including phospholipids, sphingolipids, glycolipids and cholesterol, with various functions within the cell. A growing body of evidence supports an important role for the regulation of gangliosides, which are sialic acid-containing glycosphingolipids, in epithelial mesenchymal transition (EMT). EMT is a developmental process that is necessary for the formation of most adult tissues, and is adversely linked to cancer progression. During EMT, cells acquire stem-like properties, together with increased migration and invasion of other organs.

The expression of complex gangliosides was shown to be increased and associated with invasion and metastasis in pathological conditions including several types of cancer such as melanoma, neuroblastoma, glioblastoma and breast cancer, but also endoderm-derived lung cancer [158,159]. Due to their extracellular orientation, gangliosides are involved in cell–cell and cell–matrix interactions, making tumor-associated gangliosides attractive targets for cancer therapy, perhaps in combination with immunotherapy [160]. In the context of FA, an untargeted mass spectrometry lipidomics approach identified a lipid fingerprint in FA-deficient versus FA-proficient cell lines. Consistent upregulation of the monosialodihexosyl ganglioside GM3 and several derivative gangliosides was detected. Remarkably, specific glycosphingolipids, including GM3, are known to promote EMT of human epithelial cell lines [161,162]. Indeed, blocking ganglioside accumulation with the glucosylceramide synthetase inhibitory drug zavesca/miglustat inhibited invasion by FA-deficient HNSCC cells [80].

In HNSCC cells which harbor a functional FA pathway, knockdown of FANCA, FANCD2 or FANCJ stimulated cellular conversion from an epithelial to a mesenchyme-like morphology, enhanced motility and the acquisition of invasive characteristics supported by dysregulated lipid metabolism [80]. In line with these data, in a serial engraftment mouse model of Fanca^+/+^; Tp53^−/−^; Ccnd1^OE^; HRAS^G12V^ versus Fanca^−/−^; Tp53^−/−^; Ccnd1^OE^; HRAS^G12V^, FA loss of function accelerated tumor development together with early signatures reflective of EMT, cancer stem cell transition and metastasis [163]. Thus, the application of lipidomics to FA-deficient cancer cells demonstrated that either FA DNA repair deficiency or non-canonical FA activities produce a lipid signature which promotes EMT-like HNSCC cell phenotypes and might include therapeutic targets. Further discovery of mechanism-based biomarkers and functional testing of candidate enzymes will hopefully define other targetable metabolic pathway(s) that contribute to clinical FA HNSCC phenotypes. Overall, these data link lipid dysregulation in cancer with EMT phenotypes and metastatic dissemination, and suggest that relevant enzymes and metabolites may be targetable for cancer prevention and treatment in FA individuals.

### 5.3. Tryptophan Metabolism Drives Serotonin Production in FA

A significant increase in urinary and stool tryptophan occurs after allogeneic hematopoietic stem cell transplant (HSCT) in patients with FA, but not in those without FA [37]. Tryptophan (Trp) is an essential amino acid that is used in the production of biogenic amines such as serotonin, melatonin or tryptamine, and large increases in peripheral serotonin, coupled with reduced levels of kynurenine were reported. Metabolic anomalies potentially related to tryptophan metabolism occur in children with FA, including low body mass index (BMI) and reduced stature in individuals. Metabolism of tryptophan via the kynurenine pathway can similarly stimulate the tumor micro-environment (TME), as kynurenine is a suppressive signaling molecule for Tcells and NK cells [164]. The kynurenine pathway also leads to nicotinamide via quinolinate, which can be important for the synthesis of NAD^+^ if niacin is limiting. As NAD^+^ is consumed for polyADP-ribosylation, a key covalent modification for DNA repair and transcription related enzymes [165], sustained NAD^+^ synthesis is important for continued cellular survival. Additionally, the expression of TPH1, the enzyme responsible for conversion of tryptophan to 5-OH Trp on the pathway to serotonin, was elevated in patients with FA compared with non-FA patients [37]. Taken together, these data suggest that individuals with FA preferentially metabolize tryptophan into serotonin, with markedly reduced production of kynurenine, in contrast to persons without FA.

Peripheral serotonin is typically produced in the gut and transported in platelets, serving to regulate GI motility [37]. In contrast to the marked increase in serotonin observed in serum, serotonin in stool was not increased but rather decreased after HSCT in individuals with FA, suggesting an alternative site of production [37]. Skin specimens from FA and non-FA patients were tested using immunohistochemistry and TPH1 was present in both groups, confirming the capacity for production of serotonin in the skin. However, staining for serotonin in the skin was only observed in individuals with FA, with none observed in age-matched non-FA control skin. Based on these results, the authors proposed that skin tissue damage from the transplant preparative regimen releases a surge of peripheral serotonin. Similar but lower levels of chronic tissue damage from everyday exposures may release peripheral serotonin and contribute to multiple clinical FA phenotypes including thyroid dysfunction, immune dysregulation, dyslipidemia, insulin sensitivity, abnormal BMI, and cancer susceptibility. Serotonin inhibition could potentially modify cancer risk in FA [37].

Trp metabolism also can play a role in cancer risk and cancer phenotype and may be a risk modifier in FA. Trp degradation is catalyzed by either indoleamine 2,3-dioxygenase (IDO) or tryptophan 2,3-dioxygenase (TDO) [166,167]. The kynurenine pathway has been proposed to be critical in tumor proliferation, invasion and metastatic dissemination [167,168]. Several studies have investigated IDO in HNSCC, primarily in oral and oropharyngeal cell lines. IDO is overexpressed in the tumor microenvironment of HNSCC, correlating with poor prognosis [169]. Based on these results, and after treatment with epacadostat an inhibitor of IDO1, in combination with the PD-1 inhibitors, pembrolizumab and nivolumab, HNSCC showed moderate suppression [170]. IDO1 inhibitors have been intensively investigated for cancer immunotherapy in recent years, with multiple compounds in clinical trials [167], which offer potential new therapies for FA associated HNSCC.

### 5.4. Repurposing Therapies for Disease Prevention or Therapy in FA

Therapies aimed at reducing ROS might reduce cancer risk in FA. For example, anti-oxidant therapies might counter the toxic effects of DNA crosslinkers used for cancer therapies against normal tissue, potentially resulting in improved clinical outcomes for individuals with FA or other DNA damage-associated diseases [90]. In support of this possibility, treatment with NAC restored respiration at Complex I to normal levels in FANCA-deficient lymphocytes, lymphoblasts and fibroblasts, albeit without rescue of mitochondrial morphology [113]. Beneficial effects of NAC were further tested in combination with α-lipoic acid (α-LA), a mitochondrial protective agent. The results obtained suggest that the α-LA plus NAC cocktail may be help maintain chromosome stability in FA patients [171]. Previous data also showed that the antioxidant tempol (nitroxide antioxidant and a superoxide dismutase mimetic) can delay the onset of epithelial tumorigenesis in FA mouse models [172]. Testing mitochondrially targeted therapies to mitigate metabolic dysfunction and induce a shift towards an antioxidant environment may help identify well-tolerated treatments for chronic use to improve hematopoietic stem cell function and reduce cancer susceptibility.

Quercetin, a naturally occurring flavonoid and antioxidant, has been shown to reduce ROS and alleviate insulin resistance induced by oxidative stress in FA in vitro and in vivo in animal models [173]. Moreover, quercetin has been implicated in the suppression of candidate drivers of SCC in FA, i.e., oxidative stress, NF-kappa B signaling and aldehyde mediated oncogenesis [174,175]. Clinical studies of long-term oral quercetin to improve hematopoietic stem cell function in FA patients who have not had HSCT (ClinicalTrials.gov Identifier: NCT01720147, accessed on 11 March 2022) and to prevent head and neck cancer (ClinicalTrials.gov Identifier: NCT03476330, accessed on 11 March 2022) are in progress at Cincinnati Children’s Hospital Medical Center to assess the feasibility, toxicity and pharmacokinetics of this ROS modulator in children with FA. Additional correlative studies included in the clinical trial focus on the evaluation of quercetin on the reduction of ROS, maintenance or improvement of hematopoietic stem cell (HSC) reserve, improvement of hematopoiesis (i.e., peripheral counts), insulin sensitivity/glucose tolerance genomic stability measured by micronuclei and skin integrity in FA patients.

Metformin, a guanidine derivative drug which has been used for decades to treat type II diabetes, is an additional candidate to prevent progression of marrow failure in FA. Mechanisms of action in the liver include activation of AMPK signaling, with suppression of cAMP, increased insulin sensitivity and reduced expression of gluconeogenic enzymes. By activating AMPK signaling, metformin also scavenges aldehydes via the Mannich reaction [176], thereby making it a potential candidate for the treatment of FA to prevent DNA damage. A phase II clinical trial is in progress to evaluate metformin in 15 FA patients (NCT03398824). In vitro, FA-A patient–derived fibroblasts (PD259i) treated with metformin have shown reduced levels of both chromosomal radials and breaks in FA cells [177]. It is noteworthy that other compounds with antioxidant activities have been reported to improve hematopoiesis in Fancd2^−/−^ mice, including resveratrol [178], sirtuin activator [179] and NAC [180]. However, neither resveratrol nor NAC demonstrated significant tumor suppression. The precise mode of action by which metformin and a structurally related compound, aminoguanidine, reduce DNA damage in FA cells remains unclear. Evidence supports aldehyde detoxification by both metformin and aminoguanidine, and consequent protective effects of reducing DNA damage and mitigating spontaneous chromosome breakage and radials in human FA patient–derived cells [177]. Based on the results of numerous preclinical, epidemiological and clinical studies outside the FA setting, metformin can also suppress carcinogenesis by lowering insulin levels and inhibiting the mammalian target of rapamycin (mTOR) pathway, both of which play key roles in cancer metabolism and proliferation [181,182]. Accordingly, metformin has been tested as a chemopreventive against FA SCC development in an animal model and was shown to inhibit tumor formation [177].

## 6. Human Papillomavirus Is Tropic for Keratinocytes and Causes SCC

Human Papillomaviruses (HPVs) are a group of over 100 non-enveloped viruses which cause approximately 5% of all human cancers [183]. Various HPV types can infect basal keratinocytes of the epidermis and mucosa via microwounds [184]. Cutaneous HPVs are widely present in normal human skin and contribute to skin cancer initiation in cooperation with UV radiation [185]. Mucosal HPVs are divided into two sub-types: low-risk and high-risk, depending on their ability to cause benign or malignant tumors, respectively. High-risk HPVs cause cervical, anogenital, and head and neck SCC [185]. HPV16, HPV18 and HPV31 are the most frequently detected high-risk subtypes and are transmitted primarily through sexual contact [184,186]. In most FA-competent patients, the immune system effectively targets the virus and there are no symptoms associated with infection. However, a minority of infections by high-risk HPV strains can become persistent, with integration of the HPV genome into cellular DNA, and subsequent progression to cancer [187,188,189,190]. Once infection occurs, the viral life cycle is tightly regulated by the differentiation program of infected keratinocytes, a program essential for the maintenance of stratified squamous epithelia [191]. Viral replication and protein expression are regulated by epidermal differentiation and HPV proteins are involved in replication (E1, E2), oncogenic processes (E5, E6, E7), productive genome amplification (E1, E2, E5, E6, E7), and virion production for the perpetuation of infection (E1^E4, L1, L2) [191,192,193]. Continuous expression of the oncogenes E6 and E7 is required to initiate and sustain HPV-driven carcinogenesis. These oncogenes inactivate the tumor suppressor p53 and the retinoblastoma family consisting of the Rb, p107 and p130 pocket proteins, thereby permitting cell survival in the face of excessive cellular proliferation to amplify the viral genome [194,195,196,197]. Although E6 and E7 are not by themselves sufficient to transform keratinocytes, E7 is considered the predominant oncogene and can immortalize primary human keratinocytes as a key step towards malignant transformation [194,197,198,199,200]. This E7-driven phenotype can be further enhanced by the expression of E6, which suppresses cell death as a response to inappropriate virus-induced proliferation [195,196,197,198]. The late proteins L1 and L2, which are expressed in terminally differentiated cells, form the viral capsids [192,201] and are therefore required for virion assembly, transmission and spread to other sites or hosts.

### 6.1. HPV Is Epidemiologically and Molecularly Linked to FA

Epidemiological studies have shown that persons with FA are at substantially higher risk of having oral HPV (versus non-FA controls) [202]. This suggests that apart from its role in the repair of DNA ICLs, the FA pathway suppresses HPV infection or persistence. Further studies show that FA pathway loss stimulates oncogenic phenotypes resulting from HPV infection [203], and that persons with FA are uniquely susceptible to HPV-associated cancer types. FA patients are hypersusceptible to HPV-associated mucosal warts and anogenital SCCs [204]. Crosstalk between FA and HPV has been established in laboratory and mouse models [205,206,207]; however, the association of high-risk HPV16 and HPV18 in FA HNSCC is controversial, with data both for and against important contributions to carcinogenesis [208,209,210,211,212,213]. Importantly, recent next generation sequencing data demonstrate the absence of HPV in FA HNSCC as compared to sporadic tumors, and its presence in a small subset of anogenital FA tumors [163].

Mouse models offer important additional insights into a potential role for HPV in cancers associated with FA. HPV16 E7 transgenic mice crossed with Fancd2 knockout mice show increased cell proliferation in cervical epithelium and a higher incidence of head and neck, and cervical, SCCs when compared to their Fancd2 wild type counterparts [207,214]. Interestingly, murine studies demonstrate that SCC maintenance in FA-deficient mice does not require the HPV E7 oncogene in contrast to HPV-associated malignancies in non-FA mice and persons [214]. A hit and run scenario of HPV infection and carcinogenesis might therefore be envisioned in FA. Published in vitro data using experimental models have identified an association between FA and HPV infection in promoting oncogenic phenotypes. Loss of the FA pathway in HPV positive (and negative) head and neck SCC cells led to cellular adhesion defects and stimulated invasion, consistent with aggressive progression of head and neck SCCs in patients with FA [76,77]. Furthermore, high risk HPV E6/E7 protein expression diminished FA-dependent DNA crosslink repair, and caused hypersensitivity to cisplatin [33]. Conversely, FANCD2 protein has been shown to bind to HPV genomes at viral replication foci, supporting viral episome maintenance in undifferentiated keratinocytes [33]. Finally, FA pathway loss in high-risk HPV16 or HPV31-positive differentiated epidermis stimulated DNA damage, HPV E7 protein expression, cellular proliferation, hyperplasia, excessive genome amplification and potential integration [203,215]. Overall, these data suggest FA pathway loss may cooperate with HPV on several levels, at least in a subset of FA anogenital tumors where viral sequences have been detected.

### 6.2. HPV Positive and Negative SCCs May Harbor Differential Cellular Metabolism

HPV status may determine metabolic details in individuals with FA associated HNSCC. In the general population, high risk E6 and E7 proteins are essential for the maintenance of the malignant phenotype in HPV-related SCC [216,217], and have been shown to regulate drivers of cellular metabolism and metabolic enzymes. E7 has been reported to modify glycolytic and oxidative phosphorylation enzymes to support efficient viral replication and fulfill high energy demands of cancer cells [218]. E7 is known to regulated the expression of multiple metabolic enzymes including; Hypoxia-inducible Factor-1 alpha (HIF-1α), pyruvate kinase M2 (PKM2) hexokinase 2 (HKII), phosphofructokinase-1 (PFK1), α-enolase (ENOA) and lactate dehydrogenase A (LDHA) [219,220] to stimulate glycolysis and inhibit oxidative phosphorylation. For example, PKM2 is the rate limiting enzyme in glycolysis which converts phosphoenolpyruvate (PEP) to pyruvate, catalytically active PKM2 is a tetramer but in the presence of E7 is an inactive dimer form.

Although there are limited comparative studies focused on HPV positive and negative HNSCC metabolism in individuals with FA, genes associated with metabolic pathways reported to be unique to HPV+ HNSCCs included nucleotide biosynthesis and carbohydrate metabolism [221] which is supported by increased Glucose transporter 1 (GLUT1) expression [222]. Other modifications that occur specifically in HPV+ HNSCCs include metabolism of lactate, and increased expression of COX 5B and CD147 proteins involved in respiratory metabolism and lactate export via MCT expression [222]. Despite metabolic and molecular similarities between HPV negative and positive HNSCCs, infection with HPV may shift the metabolic environment towards a Warburg-like state [223] with implications for mechanisms of transformation and HPV-specific cancer treatments [224].

## 7. Conclusions

The Fanconi anemia pathway is a classical DNA repair pathway but FA-deficient cells harbor a plethora of metabolic abnormalities which are implicated in stimulating cancer development and progression, and offer promising avenues towards prevention and treatment. In particular, the literature shows that FA is associated with diminished mitochondrial activity, particularly respiration and ATP synthesis, and an associated increase in superoxide and peroxide production that leads to protein and DNA modifications. Given the multitude of metabolic pathways associated with FA, therapies aimed at curbing ROS and aldehyde production or leveraging FA metabolic reprogramming of energy metabolism may be an attractive strategy for the development of non-clastogenic means to combat malignancies for improved clinical outcomes.

## Figures and Tables

**Table 1:** Fanconi anemia pathway genes and known associated functions.

Complex	Gene	Alternative Name	Function
Core complex	FANCA		FA core complex assembly required to mono-ubiquitinate FANCD2 and FANCI
	FANCB		FA core complex assembly required to mono-ubiquitinate FANCD2 and FANCI
	FANCC		FA core complex assembly required to mono-ubiquitinate FANCD2 and FANCI
	FANCE		FA core complex assembly required to mono-ubiquitinate FANCD2 and FANCI
	FANCF		FA core complex assembly required to mono-ubiquitinate FANCD2 and FANCI
	FANCG	XRCC9	FA core complex assembly required to mono-ubiquitinate FANCD2 and FANCI
	FANCL	POG	E3 ubiquitin ligase for FANCD2 and FANCI mono-ubiquitination
	FANCM		Recognizes ICL lesions; Recruits the FA core complex and BLM helicase; Activates ATR-Chk1 signaling; 5′-3′ DNA helicase involved in the repair of Holliday junctions and replication forks
	FANCT	UBE2T	E2 ubiquitin ligase for FANCD2 and FANCI mono-ubiquitination
ID Complex	FANCD2		Binds to FANCI; Recruits nucleases and TLS polymerases for DNA damage repair; Histone chaperone, fork protection
	FANCI		Binds FANCD2; Recruits DNA repair proteins
Downstream Effectors and DNA Repair Proteins	FANCD1	BRCA2	Controls DNA repair via HR and effector recruitment; Required for RAD51 loading and replication fork stabilization
	FANCJ	BRIP1	3′-5′ DNA helicase; Essential for DNA repair via HR and TLS
	FANCN	PALB2	Regulates BRCA2 localization to DNA damage sites; Required for DNA repair via HR
	FANCO	RAD51C	Required for DNA repair via HR
	FANCP	SLX4	Endonuclease required for the resolution of Holliday junctions; Interacts with several nucleases, including FANCQ
	FANCQ	ERCC4/XPF	DNA repair endonuclease; Functions in DNA nucleotide excision repair
	FANCR	RAD51	Recombinase required for DNA repair via HR by promoting homology search and strand invasion; Required for fork stabilization
	FANCS	BRCA1	Required for DNA repair via HR and fork stabilization; Promotes end-resection; Ubiquitin ligase activity towards histone H2A and CtIP
	FANCU	XRCC2	Required for DNA repair via HR; Stabilizes the levels of RAD51C and other RAD51 paralogs
	FANCV	REV7/MAD2L2	Required for TLS repair
	FANCW	RFWD3	E3 ligase required for DNA repair via HR; Facilitates removal of RPA and RAD51 from DNA damage sites
